# Modern Malignant Mesothelioma Manifestation

**DOI:** 10.7759/cureus.36479

**Published:** 2023-03-21

**Authors:** Abrahim N Razzak, Ali Syed, Elizabeth R Procknow, Andrea Bequest, Pinky Jha

**Affiliations:** 1 School of Medicine, Medical College of Wisconsin, Milwaukee, USA; 2 Internal Medicine, Medical College of Wisconsin, Milwaukee, USA

**Keywords:** hvac, industrial workers, industrial health, lung cancer, malignant pleural mesothelioma (mpm)

## Abstract

Malignant pleural mesothelioma (MPM) involves the uncontrolled growth of mesothelial cells that form the lining of pleural serous layers. MPM has been linked with asbestos exposure in mining and manufacturing occupations with an unforgiving prognosis of 4-18 months.

In this case report, we present a 56-year-old male with a significant past medical history of hypertension, hyperlipidemia, hepatic steatosis, and ulcerative colitis who presented to the emergency department for worsening cough, eight-pound weight loss over the previous year, night sweats, and fatigue. The patient was admitted due to right pleural effusion with lower lobe collapse seen on imaging; upon diagnostic workup including pleural biopsy, results were consistent with malignant mesothelioma of the epithelioid type. Over the course of six months post-diagnosis, the patient underwent multiple hospital admissions due to acute hypoxic respiratory failure from the segmental left upper lobe and subsegmental right upper lobe pulmonary emboli, recurrent pleural effusion, and anemia. Given the aggressive nature of MPM, the patient was determined not to be a surgical candidate and underwent palliative chemotherapy sessions until his passing.

As the patient worked in heating/ventilation/air conditioning with asbestos exposure, taking a full occupational history was crucial. MPM is relatively rare; however, the incidence has increased over the last decade due to tumor development lag time post-asbestos exposure and an increase in do-it-yourself projects. There is no cure for MPM. Multimodal treatment approaches with surgery, chemotherapy, radiotherapy, and immunotherapy have been noted in the literature.

## Introduction

Malignant mesothelioma (MM) involves the rare manifestation and growth of mesothelial cells that form the lining of visceral serous layers; the pleural layer is most commonly affected with malignant pleural mesothelioma (MPM) at 65% [1-3]. MPM has primarily been linked to asbestos exposure oftentimes associated with professions such as shipbuilding, mining, and manufacturing [1]. There are many pathophysiological theories as to what causes MPM most commonly affecting epithelioid cells: these range from pleural inflammation, activation of proto-oncogenes, free radical production, and germline BRCA 1 associated protein mutations [1,4]. There has been no evidence associating MPM with alcohol, tobacco, or dietary intake; however, smoking and asbestos exposure has been linked to lung cancer at large [1]. MPM incidence is currently at around 2,500 new cases per year in the United States compared to the incidence of lung cancer at 160,000 new cases per year in the United States, demonstrating its rarity [1]. MPM often presents clinically with chest pain, weight loss, and dyspnea with a high likelihood of pleural effusion. Even with current therapeutic advances, the prognosis for MPM is grim with inevitable death within 4 to 6 months without treatment or 15 to 18 months with treatment [1]. Early diagnosis, though difficult, can lead to surgical excision of localized MPM manifestations; however, most patients are not surgical candidates and are treated via palliative radiation [1]. In this report, we present a patient case of epithelioid type malignant mesothelioma who underwent palliative chemotherapy sessions until his passing.

This case has been previously presented at the American College of Physicians Wisconsin Chapter Scientific Meeting on September 9, 2022, at Glacier Canyon Conference Center, Wisconsin Dells, Wisconsin, United States of America.

## Case presentation

**Figure 1:**
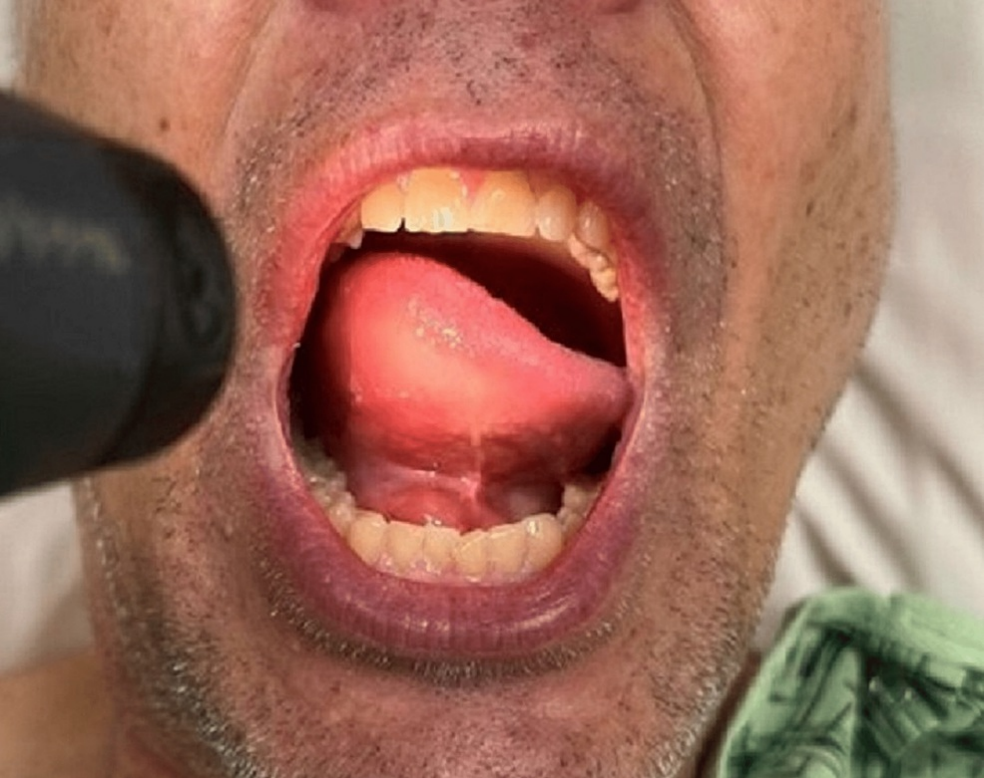
Initial clinical patient presentation with suspected ACE-inhibitor angioedema

Here, we present the case of a 56-year-old male with a past medical history of anxiety, gastrointestinal reflux disease, hypertension (HTN), hyperlipidemia (HLD), hepatic steatosis, and ulcerative colitis who presented to the emergency department (ED) with tongue swelling and a minor cough. The patient demonstrated slight swelling on the right floor of the mouth, tachycardia, and mild non-tender hepatomegaly. Blood tests were mostly unremarkable, except for thrombocytosis at 541,000/µL (normal range: 165,000-366,000/µL). As tongue swelling was the primary concern for this visit, the ED team focused on supportive treatment and airway management. Given the patient’s history, age, and medication intake of lisinopril and mercaptopurine, the diagnosis for this visit was an angiotensin-converting enzyme (ACE) inhibitor-induced angioedema and cough (Figure 1). The patient was soon discharged after intravenous supportive treatment and a small dose of lorazepam for his anxiety at the time of the visit.

**Figure 2:**
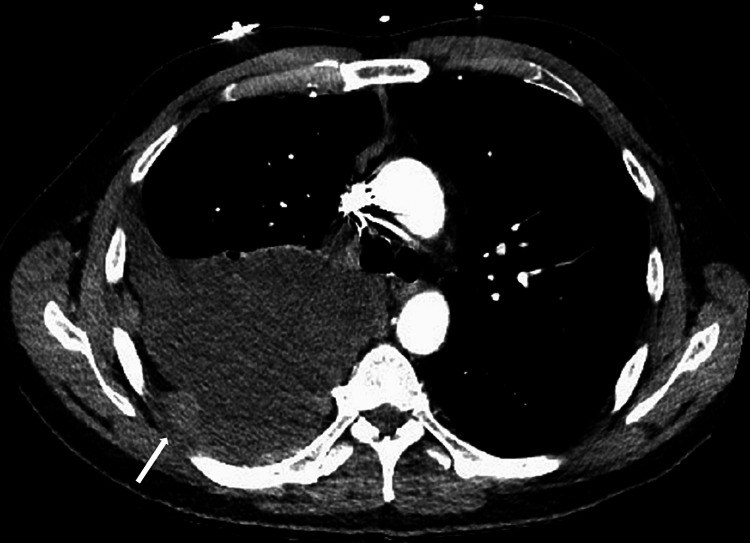
CT chest with contrast demonstrating diffuse enhancing pleural thickening and nodularity on the right; a 2.8 x 2.9cm nodular thickening noted laterally

However, the patient presented to the same ED 10 days later, now with a worsening cough disrupting activities of daily living; tachycardia (100 beats per minute) and significant dyspnea (SpO_2_ at 94%) were also present at this visit. The patient described that the cough was worsening over the past several months. He noted he was coughing up clear sputum in the morning; lying flat and exertion worsened his symptoms. While he discussed these symptoms with his primary care provider, the patient was told it was due to his history of anxiety and allergies. He reported an 8-pound weight loss over the prior year, night sweats, right upper quadrant pain with deep inspiration, and fatigue; the patient denied travel outside the country. The patient also had a smoking history of one pack year, secondhand smoking history of 20 years, and possible marijuana usage. Physical exam demonstrated significantly diminished breath sounds at the right lower and mid lung, wheezing, and elevated temperatures at 102 degrees Fahrenheit. Workup including electrocardiogram, blood tests, and computed tomography (CT) scan of the chest with contrast was conducted; this revealed elevated white blood cell counts (12.2x10^9^/L; normal range: 4.5x10^9^/L-11.0x10^9^/L) and platelet counts (621,000/µL; normal range: 165,000-366,000/µL), diffuse right pleural nodular thickening, and right pleural effusion with lower lobe collapse (Figure 2). Given these findings and concerns for sepsis or potential malignancy, the patient was admitted for in-patient care.

**Figure 3:**
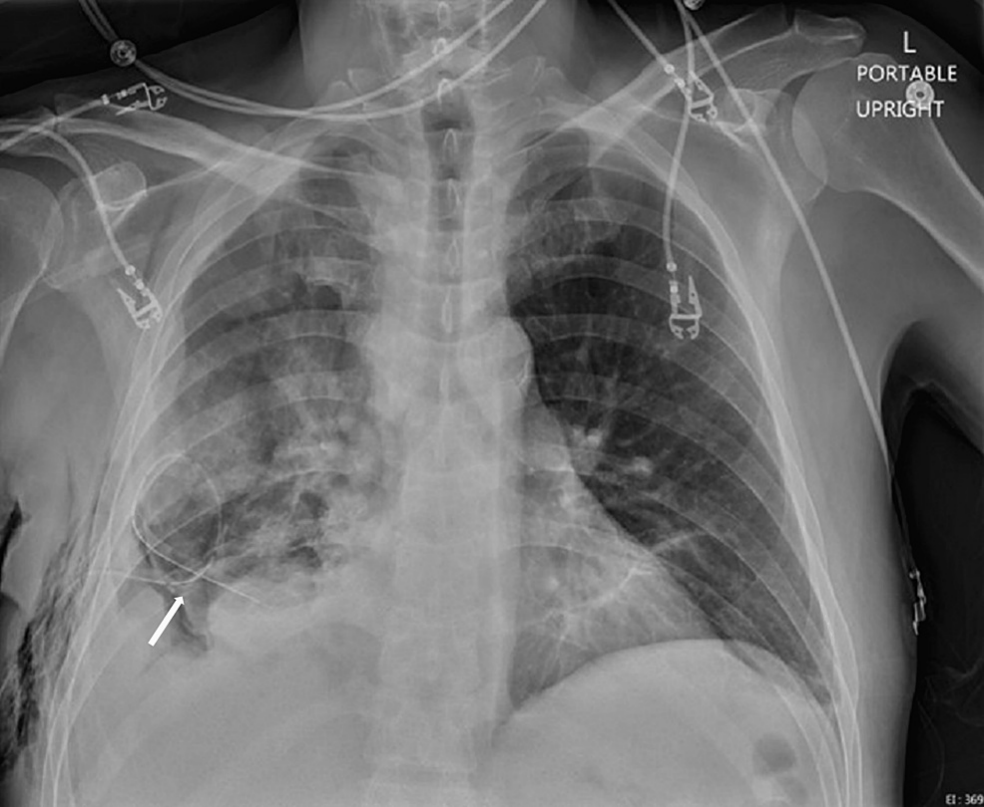
Chest x-ray taken post pleural catheter placement for pleural effusion presentation

**Figure 4:**
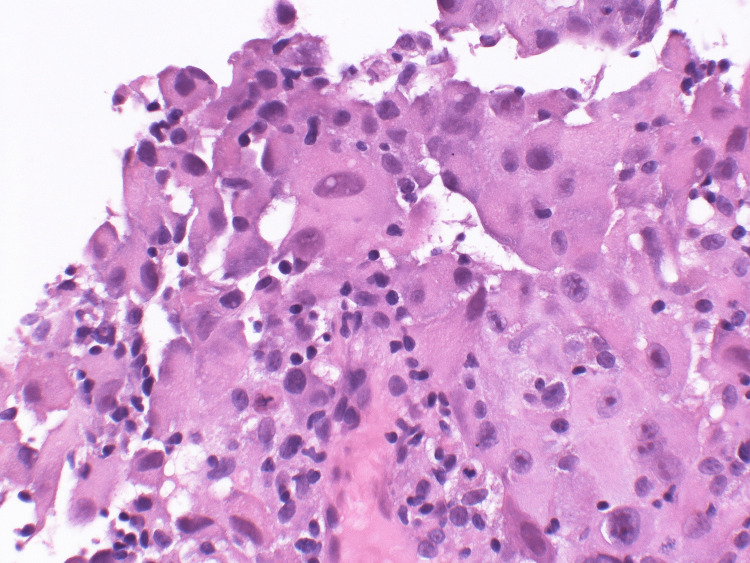
Sample pathological view of malignant mesothelioma cells of epithelioid type with hematoxylin and eosin stain This view is characterized by their close clustered round/oval-shaped adherence of the epithelioid type cells alongside identifiable nuclei. Images licensed under Attribution-ShareAlike CC-BY-SA 2.0 and permission obtained. Credits to Yale Rosen, M.D. for sample images. Source: [5]

**Figure 5:**
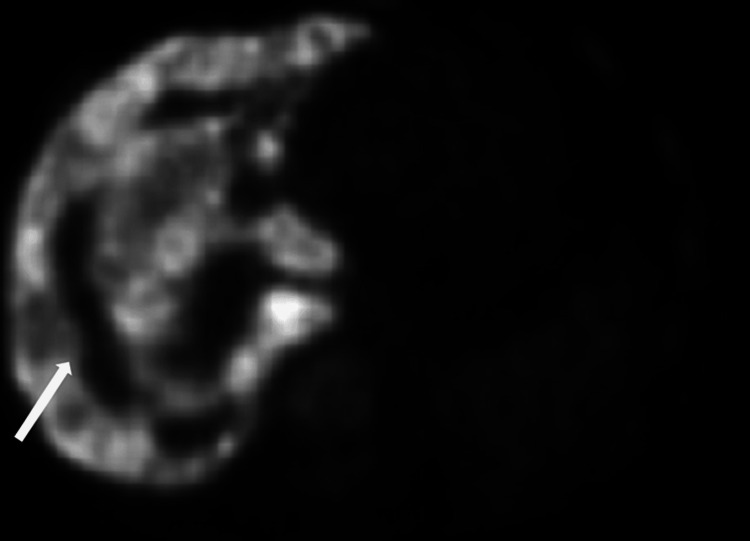
Position emission tomography scan demonstrating the persistent hypermetabolic right-sided pleural thickening along posterolateral pleural surface approximately 3cm in thickness

During his six-day stay, the patient underwent major diagnostic and therapeutic workup including thoracentesis, cytology, flow cytometry, CT chest/abdomen/pelvis with contrast, infectious workup, and pleural biopsy. Ultimately, the workup revealed that his symptoms were due to atypical mesothelial proliferation favoring reactive mesothelial hyperplasia. After discharge with negative pleural fluid cultures, the patient presented again later that month for pleuroscopy/bronchoscopy for recurrent pleural effusions thought to be secondary due to malignancy; the patient underwent a PleurX pleural catheter drainage system placement (Figure 3). Right pleural biopsy/cryobiopsy taken previously was consistent with malignant mesothelioma of the epithelioid type. Epithelioid-type mesothelioma cells are characterized by their small clustered cells in close adherence with identifiable nuclei; sample pathology images are presented (Figure 4) [5]. The patient was admitted again a week later for acute hypoxemic respiratory failure due to segmental left upper lobe and subsegmental right upper lobe pulmonary emboli, recurrent pleural effusion, and anemia, all likely secondary due to underlying malignancy. He underwent a subsequent positron emission tomography body scan which demonstrated hypermetabolic right-sided pleural soft tissue mass (Figure 5).

The patient then established care with a hematology/oncology specialist and reported prior work in heating/ventilation/air conditioning (HVAC) and maintenance for old construction sites with asbestos exposure. Given the aggressive nature of MPM and patient’s medical history, the tumor board determined patient was not a candidate for surgical intervention; palliative chemotherapy options were pursued. The patient was started on intravenous carboplatin/pemetrexed/bevacizumab systemic therapy. There was concern for immunotherapy-induced flare, should he be started onipilimumab/nivolumab due to mesalamine/mercaptopurine active treatment for ulcerative colitis, so he was not a candidate for this regimen. He completed five palliative chemotherapy sessions as described above for symptom management. Unfortunately, patient’s condition deteriorated over the course of six months, and he psychologically became depressed. The patient presented to the ED three times during these last six months due to acute on chronic right-sided chest pain; his last ED to inpatient visit workup demonstrated community acquired pneumonia with elevated neutrophilic count, hyponatremia, hypomagnesemia, and hypokalemia from poor oral intake. Per patient’s wishes, he was enrolled in hospice care and passed away at his home.

## Discussion

While MPM is a relatively rare malignancy, the incidence has increased over the last decade due to a lag time in tumor development of 30 to 50 years post-asbestos exposure, especially in industrialized nations [3,6]. Data on the incidence of MPM in developing countries is difficult to obtain, however, current data shows higher incidences in countries such as Brazil-Russia-India-China-South Africa (BRICS) where asbestos exposure precautions are less strict [7,8]. Even among industrialized countries, MM can still be attributed to asbestos exposure during home maintenance and renovation from previously built homes [9].

At this time, there is no single treatment protocol that has had a substantial impact on prolonging MPM prognosis. Currently, the focus has been a multimodal approach combining surgery, chemotherapy, and/or radiotherapy for MPM; however, the current published evidence is poor and underpowered [10]. Histological subtypes of MPM (epithelioid, sarcomatoid, and biphasic mesotheliomas with epithelioid being most common) and surgically resectable vs non-resectable tumors can also affect prognosis [3]. Epithelioid type cells have a wide variety of architectural patterns from tubulopapillary, solid, to adenomatoid; as such, there is a greater need for characterizing cytological features such as clear cell, rhabdoid, or small cell details alongside the nuclear grade and mitotic count [11]. In contrast, sarcomatoid-type cells do not have a recommended nuclear grading system and demonstrate spindle cells arranged in fascicles or haphazard patterns invading lung parenchyma with heterologous differentiation [11]. Biphasic-type cells have characters for both types, where cytokeratin expression is used to measure the spindle cell morphology of sarcomatoid-type cells [11]. There are a number of ongoing random controlled trials investigating multimodal treatments; while cancer-directed surgery for MPM may have a better survival benefit, critiques have arisen for survivorship bias and establishing definitive roles for chemotherapy, radiotherapy, and lung-sparing less invasive surgeries [12]. Immunotherapies such as pembrolizumab, tremelimumab, and durvalumab, alongside modalities such as microribonucleic acid-loaded minicells targeting epithelial growth factor receptor tumor suppression, have also been other new therapeutic modalities being looked at for MPM treatment [13,14]. The hope is these advances in new multimodal therapy and diagnostic studies can improve the troubling prognosis for MPM.

This case is a classic representation of MPM with symptoms that match the literature, such as the presence of dyspnea, weight loss, and severe coughing. Unfortunately, diagnosis is complicated and delayed due to vague symptoms presentation [3]. In this case, given the patient’s medical history of HTN on an ACE inhibitor (lisinopril), his initial ED presentation was focused on the treatment of his angioedema rather than the other symptoms. It was not until 10 days later during his second ED visit that the focus was placed on his pulmonary complaints that ultimately resulted in an eventual MPM diagnosis. As such, while it may be difficult given the demands of the healthcare systems, this case was a call for taking a complete history as opposed to a focused history for initial presentations. The case certainly highlighted the importance of taking occupational history, as the patient’s prior asbestos exposure as an HVAC worker may have led to earlier confirmation of diagnosis and treatment plans. While the patient had a smoking history of one pack-year, the literature suggests that the primary cause for MPM was driven by his occupational history. Additionally, the patient had a medical history of ulcerative colitis which complicated concerns for immunotherapy-induced flares. As noted previously, there is no single guideline established for mesothelioma immunotherapy usage. However, given that tumor flare reaction has been reported in the literature as a side effect of onipilimumab/nivolumab, careful clinical coordination for which immunotherapy to consider remained significant in this context for palliative care management [15]. Lastly, this case also identifies the lethality and poor prognosis of MPM; unfortunately, in a matter of months, the patient went from being independent with full-time work responsibilities to fully dependent and bed bound.

## Conclusions

Ultimately, while the incidence of MPM is rare compared to that of other primary lung malignancies that may overshadow similar clinical presentations, careful coordination for history taking, multi-modal treatment evaluations between healthcare teams, and understanding the prognosis and timeline for the disease continues to be crucial in modern days.
